# Temporal Gradients Controlling Embryonic Cell Cycle

**DOI:** 10.3390/biology10060513

**Published:** 2021-06-09

**Authors:** Boyang Liu, Han Zhao, Keliang Wu, Jörg Großhans

**Affiliations:** 1 Center for Reproductive Medicine, Cheeloo College of Medicine, Shandong University, Jinan 250012, China; byliu@sdu.edu.cn (B.L.); hanzh80@sdu.edu.cn (H.Z.); wukeliang@sdu.edu.cn (K.W.); 2 Key Laboratory of Reproductive Endocrinology of Ministry of Education, Shandong University, Jinan 250012, China; 3 Shandong Key Laboratory of Reproductive Medicine, Jinan 250012, China; 4 Shandong Provincial Clinical Research Center for Reproductive Health, Jinan 250012, China; 5 National Research Center for Assisted Reproductive Technology and Reproductive Genetics, Shandong University, Jinan 250012, China; 6 Department of Biology, Philipps University, 35043 Marburg, Germany

**Keywords:** embryonic development, cell cycle remodeling, developmental timing, metabolism, temporal gradient

## Abstract

**Simple Summary:**

Embryonic cells sense temporal gradients of regulatory signals to determine whether and when to proceed or remodel the cell cycle. Such a control mechanism is allowed to accurately link the cell cycle with the developmental program, including cell differentiation, morphogenesis, and gene expression. The mid-blastula transition has been a paradigm for timing in early embryogenesis in frog, fish, and fly, among others. It has been argued for decades now if the events associated with the mid-blastula transition, i.e., the onset of zygotic gene expression, remodeling of the cell cycle, and morphological changes, are determined by a control mechanism or by absolute time. Recent studies indicate that multiple independent signals and mechanisms contribute to the timing of these different processes. Here, we focus on the mechanisms for cell cycle remodeling, specifically in *Drosophila*, which relies on gradual changes of the signal over time. We discuss pathways for checkpoint activation, decay of Cdc25 protein levels, as well as depletion of deoxyribonucleotide metabolites and histone proteins. The gradual changes of these signals are linked to Cdk1 activity by readout mechanisms involving thresholds.

**Abstract:**

Cell proliferation in early embryos by rapid cell cycles and its abrupt pause after a stereotypic number of divisions present an attractive system to study the timing mechanism in general and its coordination with developmental progression. In animals with large eggs, such as *Xenopus*, zebrafish, or *Drosophila*, 11–13 very fast and synchronous cycles are followed by a pause or slowdown of the cell cycle. The stage when the cell cycle is remodeled falls together with changes in cell behavior and activation of the zygotic genome and is often referred to as mid-blastula transition. The number of fast embryonic cell cycles represents a clear and binary readout of timing. Several factors controlling the cell cycle undergo dynamics and gradual changes in activity or concentration and thus may serve as temporal gradients. Recent studies have revealed that the gradual loss of Cdc25 protein, gradual depletion of free deoxyribonucleotide metabolites, or gradual depletion of free histone proteins impinge on Cdk1 activity in a threshold-like manner. In this review, we will highlight with a focus on *Drosophila* studies our current understanding and recent findings on the generation and readout of these temporal gradients, as well as their position within the regulatory network of the embryonic cell cycle.

## 1. Introduction

Animal embryonic development is well-orchestrated in time and in space. Being composed of manifold diverse cellular and biochemical events, developmental transitions such as oocyte-to-embryo transition and maternal-to-zygotic transition are regulated by multiple, sometimes independent signals [1,2,3], which add up to precise and robust spatiotemporal regulation. With the advances of non-invasive imaging techniques, researchers are able to directly follow molecular processes and relate them to the changing morphological development in high temporal resolution [4,5,6]. Several often competing models in various experimental systems have been posited to explain the timing of developmental events [2,7,8]. Nevertheless, our understanding of timing mechanisms is still far from complete.

Embryogenesis starts with a sequence of rapid mitotic divisions, while actual growth only starts afterward. For animals with large eggs, the early cell cycles are driven by maternally supplied materials such as substrates and energy for DNA replication. The substrates include deoxyribonucleotides (dNTPs), free histone proteins, and replication factors, while the zygotic genome remains relatively quiescent [2,9]. After a species-specific number of rapid and synchronous cleavage divisions, the cell cycle is remodeled as visible by a prolonged and finally paused interphase together with a switch to a slow replication mode and a loss of synchrony [1,10]. This specific change is referred to as mid-blastula transition [2]. Concomitantly, zygotic factors gradually take over developmental control and facilitate processive morphogenesis and differentiation. Given the binary readout, cell cycle remodeling represents an excellent and sensitive assay for investigating the regulatory mechanisms of developmental timing, since both the number and the length of rapid cell cycles are on the one side easily tractable and on the other side tightly and sensitively controlled by multiple molecular and cellular timers.

The long-standing debate about an absolute or regulated timer for cell cycle pause during mid-blastula transition comes from the observation that the number of cycles depends on the DNA content, i.e., the nuclear-cytoplasmic ratio (N:C ratio) [7,11,12,13]. The egg contains a given amount of maternal cytoplasmic material, including RNAs, proteins, and metabolites, which are deposited by the female during oogenesis. While the nuclear DNA content is precisely doubled in each and every cycle, and the cytoplasm remains constant, the N:C ratio increases stepwise and may serve as a timer. After passing a threshold, N:C ratio may trigger subsequent developmental events. A central argument supporting the model is the behavior of haploid embryos, which contain half of the DNA content to start with and undergo one extra cycle before the pause. The N:C threshold is reached only after one more round of DNA replication than in diploids [7,14]. The threshold was precisely determined using aneuploid *Drosophila* embryos. Embryos with 76% to 124% of DNA content undergo the normal number of 13 cycles, while embryos with less than 70% go through one extra division similar to haploids [15,16]. Further evidence is provided by experimentally induced changes of the N:C ratio in *Xenopus* and zebrafish, leading to corresponding precocious or delayed timing of the mid-blastula transition [11,17,18]. Based on these data, the number of mitoses prior to cell cycle remodeling may not depend on an absolute time point but rather quantitatively depend on given DNA content.

Despite the simplicity of the concept, the N:C ratio comes with complications on the molecular level, as the “cytoplasm” is not a constant but includes a series of changing parameters: effective volumes of cells, nuclei cytoplasm, nuclear composition, and chromatin structure, to name some [19,20,21]. Furthermore, the N:C ratio does not only affect the cell cycle but also other developmentally controlled processes, first of all, the onset of zygotic transcription. The activation of the zygotic genome represents a key player in the timing of cell cycle remodeling and mid-blastula transition. For instance, in *Drosophila*, zygotic transcription acts upstream of cell cycle remodeling [15,22]. Less clear is the relation of N:C ratio with zygotic transcription. While some zygotic genes respond to the N:C ratio, i.e., show a delayed onset in haploids, the majority of zygotic genes are indifferent to the N:C ratio [23]. Moreover, some zygotic genes require a long interphase, i.e., they are dependent on the remodeling and prolongation of the cell cycle [24,25,26,27]. Chromatin architecture and accessibility, as well as the threshold size of cells, are the main factors for the onset of zygotic gene expression timing [20,28,29]. These observations clearly indicate that the N:C ratio is certainly one but not the only key for the timing of the mid-blastula transition.

The central components of cell cycle machinery are largely conserved among model organisms. Early embryonic cell cycles are driven by maternally provided cyclin:Cdk1 complex, whose catalytic activity determines the timing for the entry into mitosis [25,30,31,32,33]. Cyclin is synthesized in every S phase by maternally supplied mRNA and degraded in mitosis through the ubiquitin pathway [34,35,36]. The activity of Cdk1 is post-translationally regulated by an antagonistic pair of Wee1/Myt1 kinases and Cdc25 phosphatase, in which the inhibitory phosphorylation of Cdk1 T14Y15 sites is timely removed by Cdc25 in each cycle, inducing a high level of cyclin:Cdk1 activity and hence mitotic entry [3,37,38,39]. Cdk1 activity is inhibited by the activation of the DNA checkpoint, and Checkpoint kinase 1 (Chk1) is the main effector for embryonic cell cycle regulation [40].

Having stated this, we will in the following sections review molecularly defined cell cycle regulators and pathways, including replication factors, metabolites, regulatory enzymes, and free histones, some of which have been proposed to feed into the N:C ratio [27,41,42,43,44]. Undergoing gradual changes, those factors and pathways represent molecular clocks, which impinge on the Cdk1 activity, the central pacemaker of the cell cycle, and thus on timely cell cycle remodeling. The gradual changes or temporal gradients of those factors and pathways collaborate to precisely and robustly define a developmental time frame.

## 2. Activity Gradient of Cell Cycle Checkpoint

The 13 rapid and synchronous nuclear divisions in *Drosophila* are composed of S phase and M phase but lack gap phases and cytokinesis [45]. During the first 8 cycles, S phases are extremely short—only four minutes—because of an extremely fast mode of DNA replication [3,25,46]. Starting in cycle 11, when the number of nuclei reaches 2048, interphases gradually lengthen, reaching 21 min in cycle 13 before pausing in cycle 14. This lengthening depends on the DNA checkpoint with Chk1 (encoded by *grapes* in *Drosophila*) as its central regulator. *Grapes* mutant embryos keep the fast cell cycle, undergo additional cycles without any sign of the cell cycle mode change. They finally end up in a so-called mitotic catastrophe, when incompletely replicated chromosomes are subjected to mitosis [15,43,47]. Thus, both the number and the length of embryonic cell cycles are governed by the DNA checkpoint activation and Chk1 kinase activity.

At least two factors underlie the gradual activation of the DNA checkpoint. Firstly, gradually decreasing levels of dNTP metabolites become rate-limiting during rapid DNA replication due to the exponentially increasing consumption. The limited amounts of dNTP cause DNA replication stress and checkpoint activation [42]. This mechanism will be discussed in detail in Section 4. Secondly, the awakening of the zygotic genome, as seen by a gradual increase in RNA polymerase II activity, is associated with interference between replication and transcription, which leads to DNA replication stress as indicated by increased levels of single-stranded DNA and subsequent activation of the DNA checkpoint [15,22]. Eventually, in interphase 14, when the number of cortical nuclei achieves about 6000, the cell cycle switches to a slow replication mode, the DNA checkpoint is stably activated, and a full G2 phase is added [1,3].

As mentioned briefly above, the DNA replication checkpoint is obviously activated by global zygotic transcription during S phases in *Drosophila* embryonic cycles 13 and 14 [15,22]. The gradual activation correlates with de novo RNA Polymerase II recruitment and the quantity of transcriptionally engaged loci [15,48]. Inhibition of RNA Polymerase II by α-amanitin eliminates zygotic transcription and leads to an additional synchronous mitotic division prior to the cell cycle remodeling. While mutation of pioneer transcription factor *zelda* to reduce zygotic expression also shows an extra cell cycle, although with lower penetrance, embryos require an input from the zygotic genome to pause cell cycle progression [15,42,49,50,51]. One explanation is that the interference between ongoing DNA replication and the initiation of zygotic transcription results in changes in DNA replication origin usage, poised RNA Polymerase II, and recruitment of the RPA complex, thus activates the replication checkpoint [15,52]. When inducing zygotic transcription precociously, a subsequent premature cell cycle arrest takes place due to activation of the checkpoint gene [22].

Similar to *Drosophila*, the DNA checkpoint also plays a central role in vertebrate species with large eggs, such as *Xenopus* and zebrafish. *Xenopus* embryos undergo 12 fast and synchronous cleavage cycles. The interphases gradually prolong by a slowdown of DNA replication from cycle 9 onward and by gradually increasing replication stress and DNA checkpoint activation [53,54]. The cell cycle is stably remodeled in cycles 13 to 15 by processive slowing DNA replication and the addition of full gap phases [13,55,56,57,58]. Replication factors play a major role in *Xenopus* checkpoint activation. Four specific replication factors are maternally supplied in limiting amounts. The increasing number of nuclei titrates those replication factors, which restricts the number of replication initiation events and prolongs the S phase [41]. A comparable situation has been reported for zebrafish embryos. S phase lengthening and gap phase introduction are observed in cycles 10 and 11, which are accompanied by DNA checkpoint activation [11,59,60]. Mammals are quite different from oviparous animals in terms of their development pattern and regulatory machinery. The early cycles of mouse embryos contain a G1 and a G2 phase, presenting more canonical cell cycles [8,61,62].

## 3. Gradient of Cdc25/Twine Decay in *Drosophila*

The post-translational control of Cdc25 is a central mechanism in cell cycle remodeling. Of the two Cdc25 homologs in *Drosophila*, Twine and String, Twine is functionally relevant for cell cycle remodeling during the mid-blastula transition [38,49,63]. Whereas translation and degradation of maternally provided RNA are kept in balance during the early cycles. Twine’s half-life drops by an order of magnitude in interphase 14, which leads to a complete loss of the Twine protein within 20 min [4,14,64,65]. Given the need for Cdc25 dependent dephosphorylation at T14Y15, Cdk1 becomes inactive in interphase 14 with a corresponding G2 pause. The concentration profile of the Twine protein has been established in detail by Western blot with manually staged embryos and in live with a GFP-tagged Twine [4,64,66]. The half-life was determined with a switchable form, Twine-Dronpa [14]. More recently, the in vivo profile with absolute concentration was measured by fluorescence fluctuation analysis, which revealed nuclear concentrations from about 300 nM in interphase 11 to about 150 nM at the onset of cellularization, and about 41 nM as the decisive threshold of extra mitosis at 20 min of interphase 14 [4].

**Figure 1:**
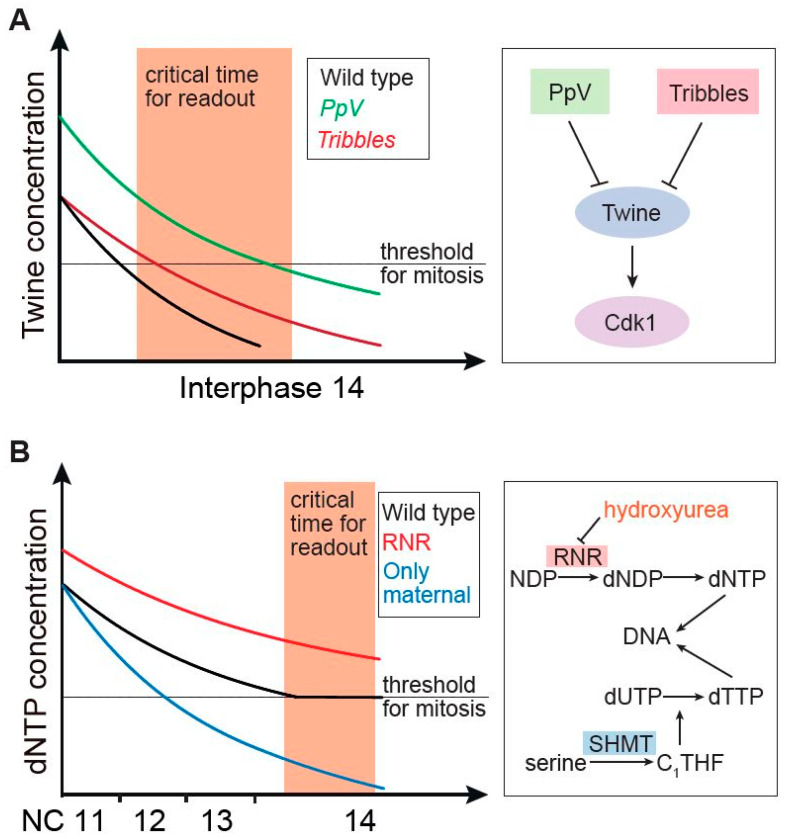
Temporal gradients of Twine protein and dNTP metabolites and their regulatory pathways. (**A**) Schematic temporal dynamics and threshold of Twine protein. (**B**) Schematic temporal dynamics and threshold of dNTP metabolites. NC, nuclear cycle. Critical time for readout indicates the time window when levels of Twine protein and dNTP metabolites reach the threshold to determine the mitotic entry.

The gradual decline of Twine protein is sensed and transformed into a binary decision by an auto-activation loop of Cdk1. Due to the positive feedback of Cdk1 on Cdc25/Twine, Cdk1 becomes fully activated if Twine is above the threshold. As soon as Twine falls below the threshold, Cdk1 will completely lose its activity. The time when Twine reaches the threshold is determined by two parameters: (1) Starting level, i.e., Twine levels at the onset of interphase 14, and (2) decay constant, i.e., the speed of Twine degradation during interphase 14. Maternally provided Protein phosphatase V (PpV) ensures low steady-state levels of Twine at the onset of interphase 14. In *PpV* mutants, Twine levels are on average 47% higher than in wild type. Twine reaches the threshold only later, even without a changed half-life, and, consequently, 30–50% of the embryos reenter mitosis. In contrast, the pseudokinase Tribbles, along with other factors, destabilizes Twine in interphase 14 without changing starting levels [4,14,64,67]. Tribbles is assumed to promote degradation of Twine, directly or indirectly. The decay time of Twine protein increases from less than 10 min in wild type to 13.5 min in *tribbles* mutants. Given the slower degradation, Twine reaches the threshold later, and, consequently, a small proportion of embryos undergo an extra cycle [4,68,69,70]. The temporal dynamics of Twine protein provide timing information to the embryo, and this input can be accurately sensed and responded to by the cells to determine if and when to enter the next mitosis (Figure 1A).

Besides *tribbles*, other specific zygotic genes are also involved in Cdk1 inactivation. For instance, Frühstart functions to inhibit mitotic entry via binding to the hydrophobic patch of CyclinA and thus suppress cyclin:Cdk1 activity. Acting as a molecular clock, Frühstart begins transcription immediately after mitosis 13, and its transcription is also dependent on the N:C ratio [23,68,71,72]. In terms of the cell cycle length, the S phase is prolonged by the introduction of delays in the replication of satellite sequences, which are composed of the blocks of repetitive DNA on the genome [25,65]. Replication repressors Rif1 and Cdc7 compose a replication timer for the satellite sequences and thus prolong the S phase in cycle 14 [73]. Moreover, in *Xenopus*, activating subunit for the Cdc7 kinase Drf1 contributes to the slowing of the S phase by Chk1 inhibition during cycle 13 [74].

## 4. Temporal Gradient of dNTP Metabolites

After fertilization, translational and metabolic pathways are activated, whereas the zygotic genome transcription is initially silent. Cytoplasmic nutrients, including catabolites used for energy production and anabolites used for biosynthesis (catabolism), provide nucleotides for RNA and DNA synthesis. The role of metabolic regulation has been little studied in controlling developmental progression thus far, but several recent reports point to an instrumental role in developmental decision and timing. With the advent of sensitive and suitable assays, dynamic profiles of many metabolites can now be measured, including gradual changes of dNTP metabolites and their role in the timing of the cell cycle. During synchronous cell cycles in early embryos, the demand for dNTPs doubles in every cycle. In principle, dNTPs are provided from two sources: (1) the maternal pool loaded during oogenesis and (2) de novo biosynthesis within the embryo after fertilization. Precise measurements revealed a gradual drop in dNTP concentrations [42]. Thus, the concentration profiles of dNTP or derivatives provide timing information, which impinges on cell cycle regulation via the DNA checkpoint.

In *Drosophila* and *Xenopus* embryos, dNTP metabolites are involved in activating the DNA checkpoint and in lengthening the cell cycle [41,42,75,76,77]. Measurements of dNTP content show that the maternal pool suffices for only a limited number of embryonic cycles. Specifically, after 11 rounds of the cleavage division, the *Drosophila* embryo contains 2048 nuclei, which correspond to incorporation into DNA of about 1.5 million dNTP per second and nucleus. The maternal pool comprises about 1.2 pmol of dCTP, 0.8 pmol of dATP, and 1.2 pmol of dTTP, which suffices for about 2700 diploid genomes. This number is reached after 12 cycles, which is one less than the actual number of nuclear cycles, indicating the need for de novo biosynthesis in the embryo [42]. Similarly, the maternal dNTP content in *Xenopus* embryos suffices for 11 cycles post-fertilization, which is also one cycle less than the normal number of cleavage cycles [78,79].

Embryos produce free dNTPs by themselves to compensate for the incorporation into DNA. A key enzyme for the regulation of dNTP synthesis is ribonucleotide reductase (RNR), which converts NDP to dNDP, and is allosterically regulated by feedback from the dNTP products. Synthesis of dNTP can be inhibited by the RNR inhibitor, hydroxyurea. The functional role of the maternal pool can be revealed by hydroxyurea treatment, which inhibits RNR in the embryo and thus de novo biosynthesis. Such embryos exclusively contain dNTPs from the female. *Xenopus* and *Drosophila* embryos treated with hydroxyurea prematurely arrest cell cycle progression and prolong the S phase, consistent with the maternal dNTP content [42,54,76,79]. Besides chemical inhibition of de novo biosynthesis, genetic aberration of the *Drosophila* metabolic enzyme serine hydroxymethyl transferase (SHMT), which is required for the single carbon (C1) metabolism and synthesis of dTMP, leads to a developmental arrest in interphase 13 [80]. Co-injection of dNTPs to hydroxyurea treated embryos or injecting dTTP to *SHMT* mutant embryos leads to timely cell cycle remodeling as in wild type, indicating that indeed limiting dNTP amounts cause DNA checkpoint activation due to replication stress, and thus the corresponding precocious cell cycle pause [42,76,79] (Figure 1B). The mechanism of dNTP-induced cell cycle arrest is dependent on Grapes/Chk1 but independent of Twine, since Twine is still present when the precocious arrest occurs [80]. In *Xenopus*, the increasing N:C ratio causes limitation of the replication factors, increasing inter-origin distance and promoting S phase elongation. The increased inter-origin distance, together with dNTP depletion, leads to activation of Chk1, resulting in further cell cycle arrest [41,81].

In addition to dNTPs, other metabolites are also found to be crucial during early embryogenesis. Energy cost has been found to be tightly associated with cell cycle timing in developing zebrafish embryos [6]. The activity of cyclin:Cdk1 complex by repeated rounds of phosphorylation and dephosphorylation through Wee1 and Cdc25 consumes the majority of energy produced in the early embryo, imposing accurate and robust time information for the developmental cell cycle [6]. Consistently, the computational model of energy cost in *Drosophila* embryogenesis shows that the polymerization of protein, RNA, and DNA requires only less than 10% of the total ATPs, suggesting that embryos use even more energy to maintain the stereotypical developmental order and timing than for the major biosynthetic processes [82]. Studies from mammals revealed other mechanisms linking metabolic and timing control. For instance, TCA cycle enzymes are required for the production of acetyl groups, which are essential for histone modifications during embryonic genome activation. Thus, pyruvate-dependent nuclear transport of TCA enzymes corresponds to the timing of genome activation in mammalian embryos [83]. TCA is also necessary for cell cycle control of early *C. elegans* embryos, as down-regulation of TCA induces cell cycle arrest at the one-cell stage [84]. Precise temporal profiles will reveal whether and how those metabolites are potentially involved in the timing of the cell cycle and developmental transitions.

## 5. Temporal Gradient of Free Histone Proteins

DNA is always present as chromatin within a cell. Linked with DNA synthesis, histone proteins assemble with naked DNA into nucleosomes and chromatin. Similar to dNTPs, the demand for histone and chromatin proteins doubles with every cycle. Histones are maternally provided or newly translated within the embryo [85]. Non-DNA-bound histones are gradually depleted with the progression of the embryonic cell cycles, given the limited capacity for de novo translation. As a basic chromatin component, histones and correspondingly nucleosomes can impinge on transcription by changing global chromatin states [86]. Observations from *Xenopus* and zebrafish embryos show that histones generally act as transcription repressors, in that histone proteins compete with transcription factors for the binding of critical regulatory elements, and excess histones above a threshold concentration repress the transcriptional activation prior to the maternal-to-zygotic transition [87,88,89,90,91]. Gradually decreasing excess histones may associate with gradually increasing zygotic transcription.

In *Drosophila*, the maternal pool of histones is sufficient to complete the first 14 embryonic cleavage cycles, while zygotic histone production is required for the progression of following cell divisions [92,93]. A reduced supply of maternal histones H2B and H3 extends the S phase of cycle 12 and leads to a precocious pause in cycle 13, one cycle earlier than the wild type. Conversely, excess histone H2B (90%) leads to accelerated cycles 13 and 14 and an extra cycle 15 in 10% of the embryos [94]. Interestingly, a replication-independent isoform of histone H3, H3.3, replaces histone H3 on chromatin during the early cell cycles concomitant with zygotic transcription [95].

Recent studies in *Drosophila* embryos investigated the mechanism for how histone protein levels impinge on cell cycle control without chromatin incorporation and challenged the model of indirect control via inhibition of zygotic transcription. In addition to the antagonism of nucleosomes and transcription, excess free histone H3 directly inhibits Chk1 kinase activity and Cdc25. The nuclear concentration of non-DNA-bound histone H3 decreases with each cycle, and that can regulate the cell cycle without chromatin incorporation [44,95]. Excess histone H3 competitively inhibits Chk1 kinase activity, and thus high levels of non-DNA-bound histone H3 suppress DNA checkpoint activation and promote cell cycle progression [44]. Therefore, histones play as signaling molecules for developmental timing independent of chromatin incorporation. The graded availability of histones in response to the increasing N:C ratio contributes to the timing of both cell cycle remodeling and zygotic transcription.

## 6. Time Scales and Readout of the Gradients

**Figure 2:**
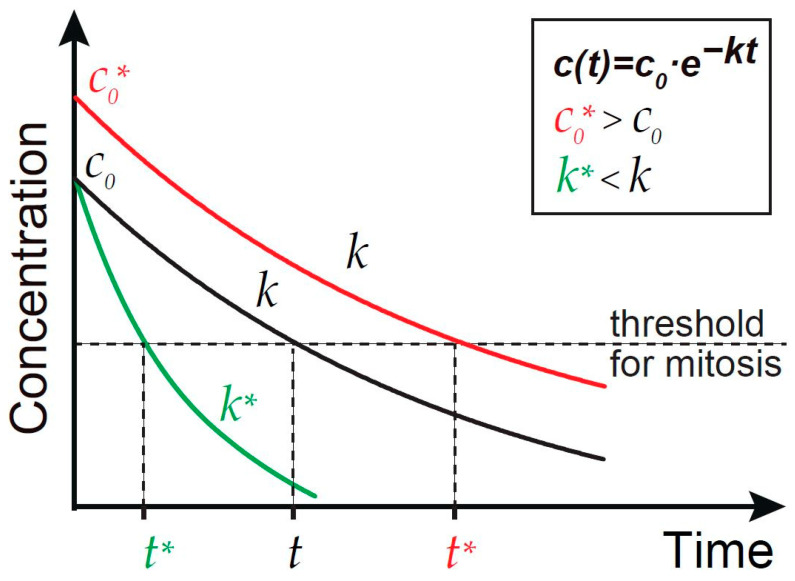
Gradients provide temporal information. Schematic time courses for wild type (black), *c*_0_* > *c*_0_ (red), and k* < *k* (green). The formula is based on exponential fitting. *c*_0_, initial concentration. *k*, exponential constant. *t*, time to reach the threshold. t*, corresponding precocious or delayed time points.

Multiple factors and pathways contribute to the timing of the processes during early embryonic development on different time scales. On the one hand, the exponentially growing DNA content represents an obvious timer on the larger scale, which is read out by binding replication factors, histones, and consumption of dNTP metabolites. These factors feed into the DNA checkpoint pathway [41,42,44]. The levels of these factors are determined by a balance of a fixed maternal contribution and of embryonic de novo biosynthesis. For example, a maternally provided dNTP pool does not suffice for all embryonic cell cycles, and thus embryonic de novo biosynthesis is required for completion of the fast cycles [75,79]. On the other hand, other timing mechanisms function on the smaller time scale of a single cycle. The graded decay of Cdc25/Twine provides precise timing within the last cycle. The Cdc25/Twine timer is triggered by the activation of zygotic transcription and less so on DNA replication cycles, and is fine-tuned by multiple factors such as speed of decay and starting levels [14]. Temporal gradients of these regulators are accurately read by embryonic cells through multiple molecular and metabolic pathways [96]. In pace with the process of early embryogenesis, several critical thresholds of titrated maternal molecular clocks are reached, together determining the timing of entry into mitosis through particular downstream pathways (Figure 2).

**Figure 3:**
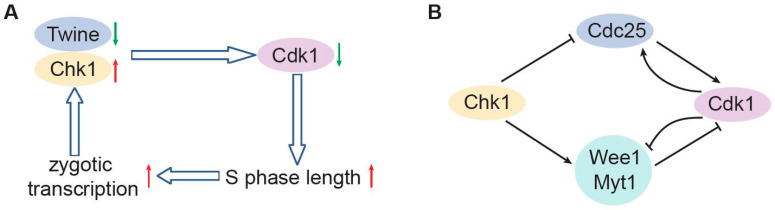
The embryonic cell cycle machinery contains positive feedback loops. (**A**) The positive feedback loop of zygotic transcription in *Drosophila* embryogenesis. (**B**) Regulatory feedback loops of Cdk1 in *Xenopus*.

The readout of the gradients involves, in each case, a positive feedback loop to yield a binary result, unambiguously and irreversibly. The positive feedback mechanisms can act indirectly involving transcription or by direct post-translational modifications to control enzymatic activity. For example, *Drosophila* zygotic transcription induces Twine down-regulation and Chk1 activation thus inhibits Cdk1 and prolongs S phases, which as a consequence promotes transcription (Figure 3A). In *Xenopus*, Cdc25 activates Cdk1 and is activated by Cdk1, forming a positive feedback loop. Similarly, the kinases Wee1/Myt1 phosphorylate T14Y15 sites of Cdk1 and thereby inactivate it, and Wee1/Myt1 can also be inactivated by Cdk1, forming a double-negative feedback loop similar to the positive feedback loop. These positive and double-negative feedback loops constitute a bistable trigger [97] (Figure 3B). In general, the intrinsically timed developmental events are initiated by thresholds reached molecular clocks, and once determined, they are ensured by multiple mechanisms of positive feedback loops to complete.

## 7. Local Response of the Gradients

The early embryonic cycles are characterized by a striking degree of synchrony, despite the physical size of some eggs in the millimeter scale. In syncytial *Drosophila* embryos, coordination can be achieved chemically via diffusible factors, thus that a “cell” is forced to behave like its neighbors. In early zebrafish embryos, cell-cell communication is maintained through persistent bridge connections that allow cells to coordinate their behavior [98]. Small differences in timing on a larger length scale become visible as division waves or cytoplasmic flows [99,100]. Such pseudo-synchrony may even have a function, as it allows overshooting spindles to ensure full chromatin segregation, for example [101]. Embryos undergoing extra mitotic division prior to cell cycle remodeling often appear only partially and incompletely, showing patched surfaces on the embryos. The timing information provided maternally and zygotically to early embryos is a global input, meaning the synchronization at early cell cycles should be maintained if all the cells share the same mitotic enter thresholds. Some inter-nuclear or inter-cellular signaling pathways ensure coherent behavior across all nuclei via communication among neighboring cells [16]. When a group of individual cells in response to temporal gradients reaches the mitotic threshold locally while the neighboring cells do not, they may enter the next mitosis and result in a patched embryo. Although active Cdk1 or its activators serve such a mechanism, additional factors may also contribute. Histone proteins and metabolites are able to diffuse and will balance differences in concentration among neighboring cells.

The demonstration that local temporal gradients acting as a molecular clock might indeed determine the mitotic entry raises the possibility that corresponding mechanisms may be present and control the timing of other developmental events and stage transitions. How local embryonic cells sense the temporal gradients accurately is still an open question that needs to be addressed in future studies. In any case, a prerequisite is robust temporal profiling of the central components by non-invasive assays with high spatiotemporal resolution. Recent advances in genetic and cellular approaches may help us further uncover the dynamics of temporal gradients and their regulatory pathways [5,102].

## 8. Conclusions and Perspectives

The early embryonic cell cycle machinery is conserved among many species, despite their diversity. For instance, in accordance with the evidence from *Drosophila*, cell cycle arrest is promoted by global zygotic transcription, and vice versa, progressively extending S phases facilitate the activation of zygotic transcription [15,22,24,27]. Consistently, the model in *Xenopus* implicates that elongating the early cycles promotes zygotic expression since longer S phase could set the pace of more transcription [41,53,103]. In contrast, however, the DNA damage checkpoint is independent of zygotic transcription in zebrafish since blocking cell cycle lengthening prior to the remodeling does not affect zygotic genome activation timing [60]. Mechanisms for the temporal coordination of cell cycle remodeling and the onset of zygotic expression appear not to be conserved. A convincing unifying model for the relation of zygotic transcription and cell cycle control across and within species has not been achieved yet. Recent findings on temporal gradients of Cdc25, dNTP metabolites, and histone proteins associated checkpoint activation and thus Cdk1 activity reveal the timing mechanism in early embryonic development and may also share a conserved regulatory role in *Drosophila* and other model organisms.

## Data Availability

Not applicable.
